# Varicella‐zoster virus infection and primary membranous nephropathy: a Mendelian randomization study

**DOI:** 10.1038/s41598-023-46517-x

**Published:** 2023-11-06

**Authors:** Lin Li, Lixin Fu, Liwen Zhang, Yanyan Feng

**Affiliations:** Department of Dermatology, Chengdu Second People’s Hospital, Chengdu, Sichuan China

**Keywords:** Genetic association study, Infectious diseases, Kidney diseases

## Abstract

Primary membranous nephropathy (MN) is a rare autoimmune cause of kidney failure. Observational studies have suggested some relationship between virus infection and primary MN, but the association remains unclear. The current study performed a two‑sample Mendelian randomization (MR) analysis to explore the causal association between varicella‐zoster virus (VZV) infection (chickenpox and shingles) and primary MN using genome‑wide association studies (GWASs) summary statistics. The exposure datasets containing chickenpox and shingles were obtained from the GWASs conducted by the 23andMe cohort. And summary‐level statistics for primary MN were used as the outcome dataset, comprising 2150 cases and 5829 controls from European Ancestry. The inverse variance weighted method was adopted as the main analysis. As a result, we found that both genetically determined chickenpox (odds ratio [95% confidential interval] = 3.61 [1.74–7.50], p = 5.59e−04) and shingles (p = 7.95e−03, odds ratio [95% confidential interval] = 2.49 [1.27–4.91]) were causally associated with an increased risk of developing primary MN. In conclusion, our MR findings provided novel genetic evidence supporting the causal effect of VZV infection on primary MN. Further studies are needed to elucidate the underlying mechanisms mediating the causal association.

## Introduction

Primary membranous nephropathy (MN) is a rare autoimmune cause of kidney failure, with an incidence estimated at about 12/million per year^1^. It is caused by circulating antibodies binding to autoantigens expressed in the podocytes (such as M-type phospholipase A2 receptor thrombospondin type 1 domain-containing 7A), and then the immune complex deposit on the outer aspect of the basement membrane, finally leading to the thickening of the glomerular capillary walls and the leakage of proteins into the urine (proteinuria)^2–4^. The etiology of primary MN remains largely unknown, while both genetic and environmental factors have been found to play important roles in the pathogenesis of primary MN^5^. Among the environmental factors, few cases have described the occurrence of nephropathy triggered by Varicella zoster virus (VZV) infection^6^.

VZV infection is common worldwide. The global new VZV infection cases in 2019 were over 83 million, and the global age‐standardized incidence of VZV infection has continued to increase in the past 3 decades^7^. Primary VZV infection causes chickenpox, and then VZV becomes latent in ganglionic neurons^8^. As cellular immunity to VZV shrinks with age or in immuno-suppressed individuals, VZV would become reactivated, which causes herpes zoster (shingles) in tissues innervated by the involved neurons, and ultimately leads to persistent radicular pain (postherpetic neuralgia)^8^. VZV infection has been found to be a trigger for several autoimmune diseases, such as systemic lupus erythematosus (SLE)^9^ and multiple sclerosis (MS)^10^, while little is known about the relationship between VZV infection and primary MN.

**Figure 1:**
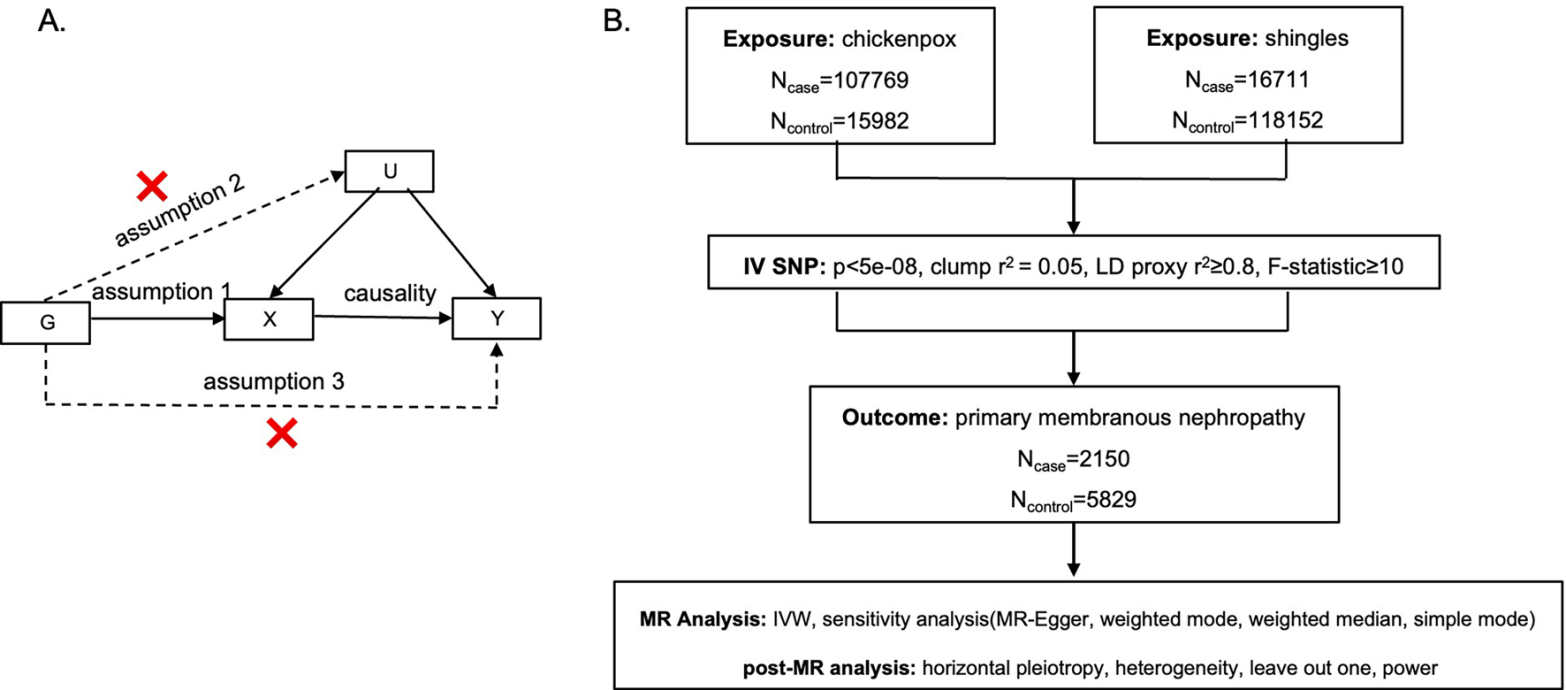
(**A**) Schematic diagram of the hypothetical relationship between genetic variant (G), exposure (X), and outcome (Y), in the presence of unobserved confounding factors (U). Solid arrows represent allowed associations between the variables, while dashed lines represent associations that need to be avoided for G to qualify as a robust instrumental variable (IV). (**B**) Diagram of the process for the 2-sample MR analysis.

Mendelian Randomization (MR) is an analysis method for non-experimental studies in genetic epidemiology, which utilizes genetic variants (G) strongly associated with the exposure (X) as instrumental variables (IVs) to assess the causal effect of exposures (X) on outcomes (Y) (Fig. 1A)^11^. For MR, the key step of MR is selecting eligible IVs (G), which are required to meet 3 assumptions: assumption 1 is that IVs (G) should be strongly related to the exposure (X); assumption 2 is that IVs(G) should not have direct associations with any other confounding factors (U) that may affect the outcome, and assumption 3 is that IVs (G) should not be directly related to the outcome (Y)^12^. Only if these assumptions are fulfilled, a causal effect of X on Y can be estimated using MR, taking G into account. There are distinct advantages of MR over observation studies. Firstly, assumption 2 and the test of horizontal pleiotropy in MR can avoid confounding factors^11^. Moreover, the direction of G → X → Y ensures the direction of the causal effect and can avoid reverse causation^11^. MR has been applied to find the causal effect of VZV infection on MS^13^ and Alzheimer’s disease (AD)^14^.

In the current study, we aimed to apply the two‐sample MR approach to investigate the causal link between VZV infection (exposure) and primary MN (outcome). Briefly, single nucleotide polymorphisms (SNPs) strongly associated with chickenpox and shingles^15^ were used as IVs to estimate the causal effect of VZV infection on primary MN^16^.

## Methods

The flowchart of the study was described in Fig. 1B.

### GWAS datasets

GWASs require much larger sample sizes to achieve adequate statistical power, which guarantees the success of detecting loci strongly associated with human complex diseases^17^, so we chose the largest GWASs to conduct the MR analyses. Chickenpox and shingles are both caused by the infection of VZV, therefore, we chose both chickenpox and shingles as exposure phenotypes to represent VZV infection. And primary MN was used as the outcome phenotype. Summary statistics of chickenpox and shingles were downloaded from the chickenpox and shingles GWASs conducted by the 23andMe cohort, which included 107,769 cases and 15,982 controls for chickenpox, and 16,711 cases and 118,152 controls for shingles, with more than 97% of the participants from European ancestry^15^. Summary‐level statistics for primary MN were obtained from the GWAS meta-analysis, comprising 2150 primary MN cases and 5829 controls^16^. All primary MN cases used in this study were defined by a kidney biopsy diagnosis of idiopathic MN^16^. Any suspected secondary cases due to drugs, malignancy, infection, or autoimmune diseases were excluded^16^. All datasets mentioned above were obtained from publicly available repositories. Sample collection, genotyping and quality control were conducted by the researchers who published the original publications, and the detailed methods can be found as referenced^15,16^. Detailed information on the records was listed in Supplementary Table 1.

### Identification of eligible IVs

Eligible IVs for MR should meet 3 assumptions^12^. To meet assumption 1 (IVs should be strongly related to the exposure) and assumption 3 (IVs should not be directly related to the outcome), SNPs that were significantly associated with the exposure but not with the outcome on a genome-wide basis (p < 5.0e−08) and had a Cragg–Donald F-statistic > 10 were selected as strong IVs^18^. However, assumption 2 (IVs should not have direct associations with any other confounding factors) was difficult in the IV selection stage because confounding factors were hard to define, therefore, it was calculated as horizontal pleiotropy in the post-MR analysis^12^.

### Two-sample MR analysis

To ensure enough number of IVs, independent IVs were set at a threshold of linkage disequilibrium (LD) at r^2^ = 0.05 within the window of 10 mega-base pairs to avoid double counting and biased causal effect estimates. Next, the IVs were extracted from the outcome trait and were harmonized in both exposure and outcome GWAS datasets. In this step, palindromic SNPs with intermediate allele frequency were removed. Moreover, if a particular requested SNP was not present in the outcome GWAS dataset, then an SNP (proxy) that is in LD with the requested SNP (target) will be searched, which was defined using 1000 genomes European sample data (r^2^ ≥ 0.8). After these steps, the MR effect can be estimated. The inverse variance weighted (IVW) method was performed as the main analysis, which is the most efficient analysis method with valid IVs because it accounts for heterogeneity in the variant-specific causal estimates^19^. Moreover, additional sensitivity analyses including the simple mode, weighted mode, weighted median, and MR-Egger regression methods, were further conducted to assess the robustness of the findings^19^. And the leave-one-out analysis was conducted within the IVW method to assess the influence of individual variants on the observed association. Furthermore, post-MR analyses including Cochran’s *Q* test for heterogeneity and MR Egger Intercept test for horizontal pleiotropy were also performed^20^. Lastly, we computed the proportion of variance in the phenotype explained by IVs and calculated the statistical power for the MR study with a two-sided type-I error rate α = 0.05^21^. The main statistical analyses were conducted using TwoSampleMR (v.0.5.6) in the R package (V.4.2.2)^22^. The study was performed according to the STROBE MR guideline^23^.

## Results

**Figure 2:**
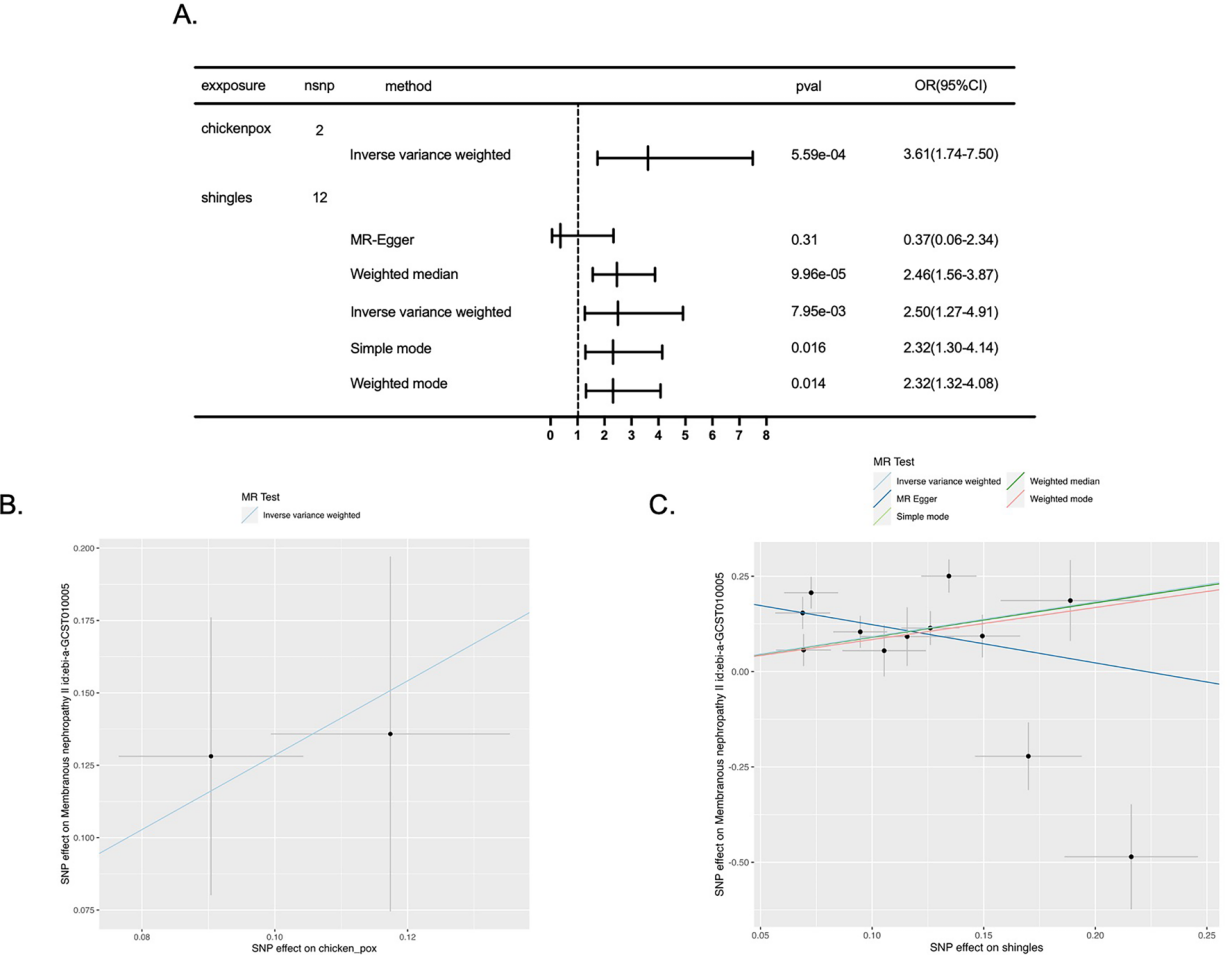
Genetic causation of VZV infection on primary MN. (**A**) Forest plot summarizing the effect of chickenpox on MN and the effect of shingles on primary MN (lower panel); *OR* odds ratio, *CI* confidential interval, *IVW* inverse variance weighted. (**B**) Scatter plot of the effect of chickenpox on primary MN; (**C**) Scatter plot of the effect of shingles on primary MN.

We first explored the causal association between chickenpox and primary MN. After harmonization, there were only 2 eligible IVs for chickenpox (Supplementary Table 2). Using the IVW method, we found that genetically predicted chickenpox was significantly associated with an increased risk of developing primary MN (p = 5.59e−04, OR [95% CI] = 3.61 [1.74–7.50]) (Fig. 2A,B). The Cochran’s *Q* test indicated no heterogeneity across the IVs (p = 0.726) (Table 1). While sensitivity analysis, leave-out-one analysis, and horizontal pleiotropy test were not available because of the insufficient IVs. The power of assessing the causality of chickenpox on primary MN was 0.72.

**Table 1:** Results of horizontal pleiotropy test, test of heterogeneity and power.

Exposure	Outcome	Horizontal pleiotropy			Test of heterogeneity (MR-Egger)			Test of heterogeneity (IVW)			Power
		Egger_intercept	se	pval	Q	Q_df	Q_pval	Q	Q_df	Q_pval	
Chickenpox	Primary MN	NA	NA	NA	NA	NA	NA	0.12	1	0.726	0.72
Shingles	Primary MN	0.223	0.104	0.06	46.87	10	9.94e−07	68.37	11	2.48e−10	1.00

We then explored the causal association between shingles and primary MN. As for shingles, there were 12 eligible IVs after harmonization (Supplementary Table 3). With the IVW method, we found that genetically determined shingles was also causally associated with an increased risk of developing primary MN (p = 7.95e−03, OR [95% CI] = 2.49 [1.27–4.91]). And the results were further supported by sensitivity analyses including weighted median (p = 9.96e−05, OR [95% CI] = 2.46 [1.56–3.87]), simple mode (p = 0.016, OR [95% CI] = 2.31 [1.29–4.14], and weighted mode (p = 0.014, OR [95% CI] = 2.31 [1.31–4.07]). While the MR-Egger analysis did not reach statistical significance (p = 0.31) (Fig. 2A,C). The leave-one-out results suggested that the causal effect was not driven by a single IV (Supplementary Fig. 1). Although Cochran’s *Q* test detected some heterogeneity among the IVs (Table 1), the intercept of MR-Egger was not significantly deviated from zero, suggesting no apparent horizontal pleiotropy (Egger intercept = 0.223, p = 0.06). The power of assessing the causality of shingles on primary MN was 1.00.

## Discussion

The viral etiology of primary MN has been less commonly studied. To the best of our knowledge, this is the first MR analysis to examine the causal association between VZV infection and primary MN. Our results support the significant causal effect of VZV infection on primary MN. The result is less prone to reverse causality and confounding bias than many previous conventional observational studies. Moreover, we adopted both chickenpox and shingles to represent VZV infection, which further confirmed the robustness of our findings that VZV infection is causally associated with primary MN.

However, in the MR results estimating shingles’ causal effect on primary MN, we noticed that the trend of MR-Egger result (OR < 1.00 but not significant) was different from the trend in the main IVW analysis (OR > 1.00 and significant) (Fig. 2A,C), which might be caused by that MR-Egger allows the IVs to have pleiotropic effects and yields less precise estimates owing to a power penalty^24^. Moreover, the other 3 sensitivity analyses supported the IVW results. Therefore, our results provided reliable evidence that shingles was causally associated with a higher risk of developing primary MN.

Primary MN is an autoimmune disease. The pathophysiological mechanism of primary MN is that the circulating antibodies bind to autoantigens expressed in the podocytes, leading to the thickening of the glomerular capillary walls and the leakage of proteins into the urine (proteinuria)^3,4^. Viruses are known to trigger autoimmunity via mechanisms such as molecular mimicry, where a foreign antigen shares sequence or structural similarities with self-antigens^25,26^. Therefore, it can be speculated that after VZV infection, the immune system might mistake the VZVs as antigens, and the antigen–antibody complex will deposit on the basement membrane and ultimately lead to the development^13^, while further functional studies are needed. On the other hand, the innate immune response to VZV might trigger inflammatory cytokine secretion and/or cell death, which further involves the pathogenesis of primary MN^27^. However, further studies were warranted to clarify how VZV infection is involved in the development of primary MN.

VZV, also known as human herpesvirus 3, is a ubiquitous alphaherpesvirus with a double-stranded DNA genome^28^. VZV infection is very common in the general population, and previous studies have shown that the incidence ranges from 13 to 16 cases per 1000 persons per year and the global age‐standardized incidence of VZV infection has continued to increase in the past 3 decades^7,8^. Moreover, VZV infection is highly communicable, and spreads by the air-borne route, with an extraordinarily high transmission rate^8^. Fortunately, antiviral drugs and vaccines against both VZV are available and are effective in treating and preventing VZV-induced disease^29,30^. Therefore, our MR results indicated that prevention of VZV infection, such as administering the vaccine, might decrease the risk of developing primary MN. However, considering that VZV is common while primary MN is pretty rare, other factors such as immune response differences between individuals should also be noted, which needs further investigation. 

There were several advantages of the current study. Firstly, we used SNPs strongly associated with the exposure (chickenpox and shingles) as valid IVs to calculate the causal association from exposure to outcome (primary MN), therefore, it ensures the direction of the causal effect and can avoid reverse causation. Secondly, the test of horizontal pleiotropy can ensure the causal association between the exposure and the outcome without the effects of confounding factors. Thirdly, we applied both chickenpox and shingles as phenotypes to represent VZV infection, which confirmed the robustness of our findings.

However, some limitations should be acknowledged in the current study. First, there were only 2 IVs used in analyzing the causal effect of chickenpox on primary MN, the IV-based heritability could only explain a small proportion of variance, and the statistical power of assessing the causality of chickenpox on primary MN was slightly low (0.72); therefore, GWASs with a larger sample size would be helpful. Secondly, chickenpox and shingles cases were determined by self-reported history, without confirmation of laboratory test results, which might bias the GWAS results. Therefore, GWAS conducted among laboratory-confirmed VZV infection cases was needed. Thirdly, the datasets used in the current study were all derived from the European population, more studies from other ethnic groups are needed to confirm the results. Last but not least, the underlying mechanisms mediating the causal association between VZV infection and primary MN were not investigated, and further functional studies were needed.

## Conclusion

In conclusion, our MR findings using GWAS summary statistics provided novel genetic evidence indicating the causal effect of VZV infection on primary MN. Therefore, prevention of VZV infection, such as administering the vaccine, might decrease the risk of developing primary MN. Further studies are needed to elucidate the underlying mechanisms mediating the causal association.

### Supplementary Information

Supplementary Figure 1.

Supplementary Table 1.

## Data Availability

The GWAS datasets used were available from the original studies. The data analyzed during the current study are available from the corresponding author upon reasonable request.
