# Supplementary material for: Varicella‐zoster virus infection and primary membranous nephropathy: a Mendelian randomization study
Source: Sci Rep. 2023 Nov 6;13:19212. doi: 10.1038/s41598-023-46517-x (PMC10628161; doi:10.1038/s41598-023-46517-x)
Supplement: Supplementary file 1 — Supplementary Figure 1. [file 41598_2023_46517_MOESM1_ESM.pdf]

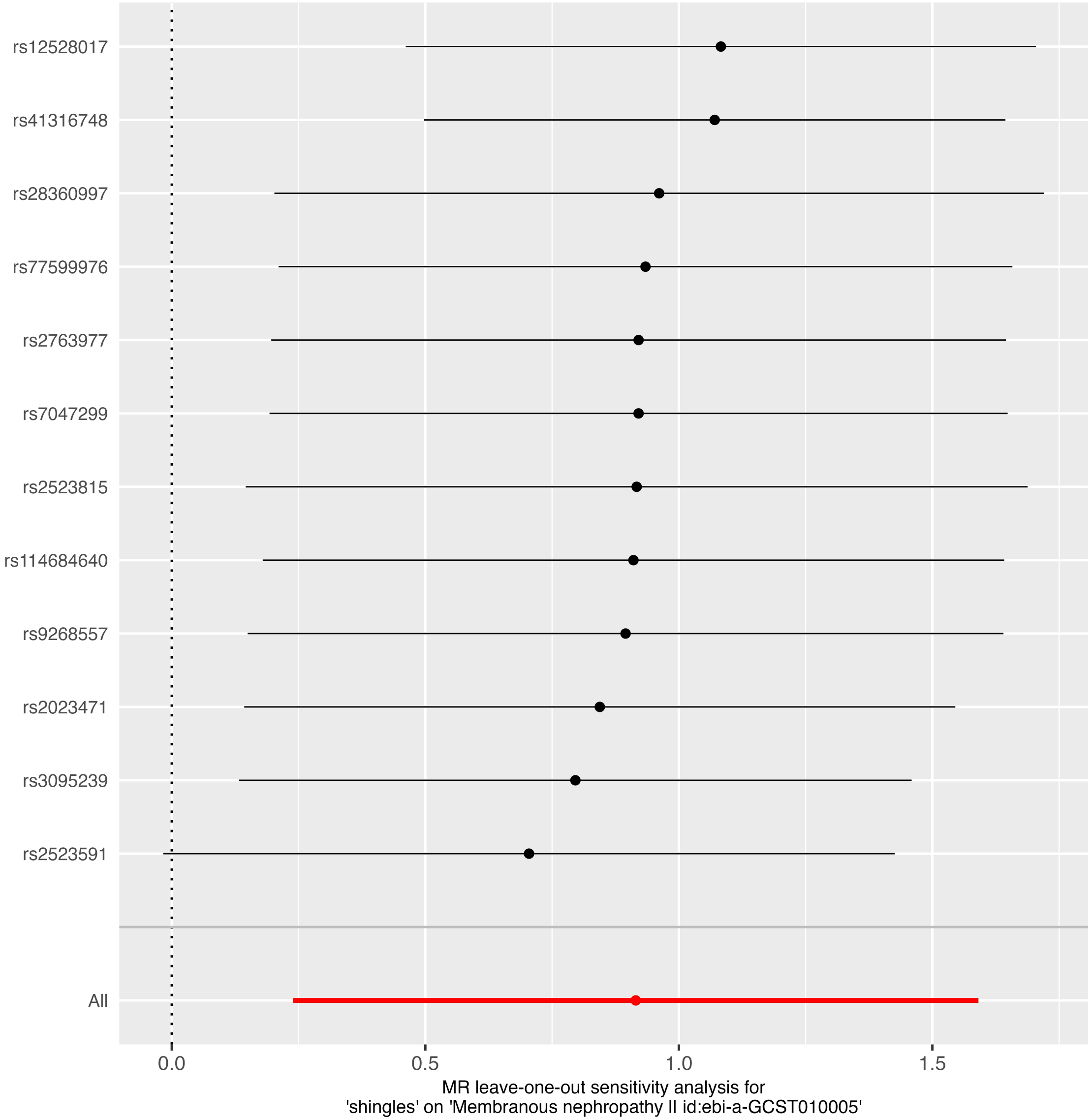

Supplementary Figure 1: MR leave-one-out sensitivity analysis suggested that shingles' causal effect on primary MN was not driven by a single IV.
